# Upstream therapy with statin and recurrence of atrial fibrillation after electrical cardioversion. Review of the literature and meta-analysis

**DOI:** 10.1186/1471-2261-12-107

**Published:** 2012-11-21

**Authors:** Lorenzo Loffredo, Francesco Angelico, Ludovica Perri, Francesco Violi

**Affiliations:** 1 I Clinica Medica, Sapienza University of Rome, Viale del Policlinico 155, Rome, 00161, Italy

**Keywords:** Atrial fibrillation, Statin, Electrical cardioversion

## Abstract

**Background:**

Atrial fibrillation (AF) is the most common sustained arrhythmia observed in clinical practice. Electrical cardioversion (EC) is commonly used to restore and maintain sinus rhythm but it is characterized by high rate of recurrences. Several trials analyzed the effects of statins to reduce the recurrences in AF with contradictory results.

**Methods:**

We performed a meta-analysis of the interventional trials with statins in patients with persistent AF to evaluate recurrences after EC. Only randomized controlled trials were included in the analysis. Data sources included: Medline, ISI Web of Science, SCOPUS and Cochrane database (up to June 2012). Data extraction was performed by three authors. Study-specific odds ratios (ORs) were combined using fixed-effects model.

**Results:**

Six studies with 515 patients were included in the analysis. Statins used in the selected trials were: atorvastatin (at dosages ranging from 10 to 80 mg/day), pravastatin (40 mg/day) and rosuvastatin (20 mg/day). AF recurrence after EC occurred in 108/258 (41.8%) of patients treated with statins and in 132/257 (51.3%) patients not on treatment with statins. Compared with control, recurrences were significantly reduced with statin treatment (O.R.: 0.662; 95% C.I., 0.45-0.96; p=0.03); statin treatment was associated with an absolute risk reduction of 0.095 and a number needed to treat of 11.

**Conclusions:**

This review suggests that statin therapy was significantly associated with a decreased risk of recurrence in patients with persistent AF after EC.

## Background

Atrial fibrillation (AF) is the most common sustained arrhythmia observed in clinical practice with prevalence increasing with age [1]. Patients with AF have about 5-fold increase of stroke risk, which is prevalently dependent on thrombosis occurring in the left atrium or left atrium appendage [2]. Restoration of sinus rhythm in patients with AF is a strategy to prevent the cardiovascular and thromboembolic complications of this arrhythmia [3].

Persistent AF is one presentation of the disease. AHA guidelines defined persistent AF when the arrhythmia sustains beyond 7 days [1] and usually terminates with pharmacological therapy or direct-current electrical cardioversion (EC) [1].

Sinus rhythm restoration in AF is not associated to reduction of thromboembolic complications and mortality [4-6]. However, the maintenance of sinus rhythm, in patients with AF, gives potential benefits such as the prevention of electrical and structural remodeling of the atria, improved haemodynamic function, amelioration of symptoms, and improvement for quality life [7].

EC is commonly used, with antiarrhythmic drugs, to restore and maintain sinus rhythm [1]. These therapies are limited by low efficacy [8]; one week after successful EC, about 25% of patients will experience a recurrence of the disease [8]. Besides research efforts to improve the efficacy of antiarrhythmic agents, there is a growing interest to the "upstream" therapy of AF [9,10]. Potential upstream therapies, which seek to inhibit the formation and evolution of the substrate for AF, include statins and angiotensin-converting enzyme inhibitors. Statins possess anti-inflammatory and antioxidant effects which can counteract inflammatory and oxidative stress pathways which are believed to contribute to the pathogenesis of AF [11,12].

The relationship between statin therapy and AF recurrence in patients with AF has been evaluated by several meta-analyses, which provided, however, conflicting results [13-16]. These meta-analyses, however, did not specifically investigate if statins reduce recurrence in patients with persistent AF undergoing EC as different clinical settings associated with AF were included in the meta-analyses. Therefore, the main objective of our study was to systematically review and analyze the effect of statin therapy on recurrence of AF after EC.

## Methods

### Eligibility criteria

#### Types of studies

Randomized clinical trials (RCTs) studying the effect of statins on recurrence of AF. No language, publication date, or publication status restrictions were imposed.

#### Types of participants

Patients of any age, with persistent AF treated with EC, were considered. Patients were excluded from this review if AF was treated with surgical interventions.

#### Types of outcome measures

The rationale of this review was to analyze the clinical effectiveness of statins to reduce the recurrences after EC in patients with persistent AF.

Because EC is unsuccessful at converting AF to sinus rhythm in some patients we performed a per-protocol analysis, including only the patients who restored to normal rhythm.

### Information sources

The studies were identified by searching electronic databases. This search was applied to Medline, ISI Web of Science, SCOPUS and Cochrane database. The last search was run on 9 June 2012. Reference lists of all studies included in the present systematic review were screened for potential additional eligible studies.

### Search

Studies were identified by searching Medline, ISI Web of Science, SCOPUS and Cochrane Database by crossing each of the following keywords:

statin

recurrence

atrial fibrillation

electrical cardioversion

randomized controlled trials

### Study selection

Two authors (L.L., L.P.) independently reviewed all selected titles and abstracts. Studies were excluded if the title and/or abstract was not appropriate for the aim of our review. Full texts were subsequently obtained for eligible studies or when the relevance of an article could not be excluded with certainty. Disagreement was resolved by consensus and by opinion of a third reviewer (F.V.), if necessary.

Studies not including a control group drawn from the same population, animal studies, or trials that exclusively reported other clinical outcomes were excluded. Case reports, editorials, commentaries, letters, review articles, guidelines or secondary prevention trials were also excluded from the analysis.

### Data extraction and quality assessment

For RCTs we planned quality assessment (Table 1) by means of Jadad’s scale [17] which evaluates the following three study characteristics: method of randomization, method of blinding, and follow-up. To stratify RCTs, we applied the following cut-offs: a total of five points defined high quality studies; three and four points defined medium quality studies; two or less points defined low quality studies.

**Table 1:** Characteristics of the studies included in the metanalysis

**Author**	**Number of patients and mean Age (years)**	**Males (%) statin/control group**	**Intervention**	**Follow-up**	**Total cholesterol (TC) and LDL cholesterol values before and after treatment with statin,mean mg/dl and % reduction**	**Co-morbidities (%) statin/control group**	**Concurrent Medications (%) statin/control group**	**Placebo-controlled/Double blind**	**Unsuccessful Cardioversion statin/control group (n)**	**Mean Left Atrial diameter (mm) Statin/control group**	**Jadad Score**
*Almroth 2009*[21]	234 (65±10)	76/74	Atorvastatin 80 mg/day (started at least 14 days before EC)	30 days	TC:202–136 (−32.6%)	H:47/48 D:7/10 CAD:4/4	B:87/80 C:14/16 DG:16/20	YES/YES	8/13	44/43	5
					LDL:123–65 (−52.8%)						
*Ozaydin 2006*[22]	48 (62±11)	71/50	Atorvastatin 10 mg/day (started 48 hours before EC)	3 months	TC:179–151 (−15.6%)	H:42/38 D:21/25	B:33/25 C:21/42 DG:4/4 A:8/4 P:8/8	NO/NO	0	47/43	2
					LDL:109–95 (−12.8%)						
*Xia 2009*[23]	64 (61±8)	69/63	Rosuvastatin 10 mg/day (started 48 h before EC)	3 months	TC:159–131 (−17.6%)	-	B:38/31 C:31/34 A:22/28 DG:13/6	NO/NO	0	41/41	1
					LDL:97–83 (−14.4%)						
*Tveit 2004*[20]	114 (68±10)	79/75	Pravastatin 40 mg/day (started 3 weeks before EC)	6 weeks	Mean n.s.	H:42/44 D:9/4 COPD:11/11 CHD:9/12	B:67/65 C:30/28 DG:25/23 F:6/4 A:2/4	NO/NO	11/11	45/43	3
					TC:-22%						
					LDL:-33%						
*Demir 2011*[25]	44 (61±10)	48/44	Atorvastatin 40 mg/day (started 3 weeks before EC)	2 months	TC:174–129 (−25.8%)	H:74/52 D:8/4 S:17/17 CHD:21/13	A:91/87 P:4/8 C:22/26	NO/NO	1/1	42/43	1
					LDL:112–62 (−44.6%)						
*Negi 2011*[24]	64 (55±12)	82/84	Atorvastatin 80 mg/day (started at randomization: 0–7 days before EC)	12 months	TC:183–142 (−22.4%)	H:52/49 D:12/3.2 S:24/16 CHD:12/13	B: 49/68	YES/YES	-	46/46	4

*H*, hypertension; *D*, diabetes mellitus; *S*: smoking; *COPD*, chronic obstructive pulmonary disease; *CHD*, coronary heart disease; *B*, beta blockers; *C*, calcium channel blockers; *DG*, Digitoxin; *F*, Flecainide; *A*, Amiodarone; *P*, Propafenone; *n.s*., not specified.

This review was conducted and reported according to the PRISMA (Preferred Reporting Items for Systematic Reviews and Meta-Analysis) Statement issued in 2009 [18].

### Statistical analysis

To evaluate the effect of statins on recurrence of AF after EC, we allocated the results of each randomized controlled trial as dichotomous frequency data. Odds ratios (ORs) and 95% confidence intervals (CIs) were calculated. These data were pooled using a fixed-effects model (the Mantel–Haenszel method) [19]. Statistical heterogeneity was evaluated using the *I*^2^ statistic, which assesses the appropriateness of pooling the individual study results [17]. The *I*^2^ value provides an estimate of the amount of variance across studies due to heterogeneity rather than chance. *I*^2^ <30% indicates mild heterogeneity, 30–50% moderate, and >50% severe heterogeneity.

The software Comprehensive Meta Analysis (version 2.2.064, USA, 2011) supported the analysis.

## Results

**Figure 1:**
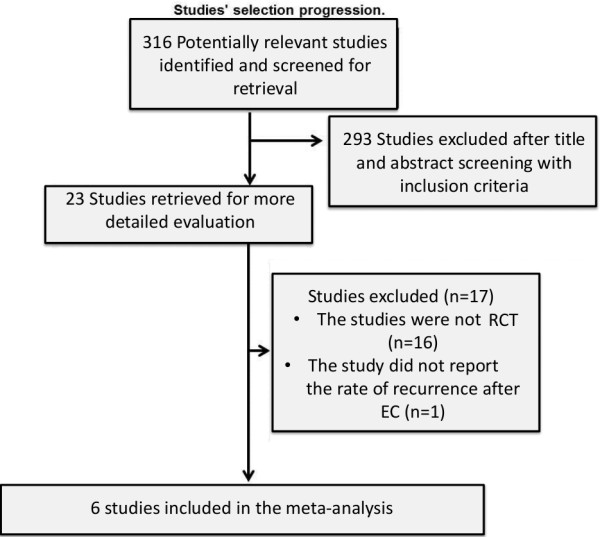
Flow diagram of study selection.

The search provided a total of 316 citations. Of these, 293 studies were discarded after reviewing the titles and abstracts because it appeared that these papers clearly did not meet the selection criteria (Figure 1).

A total of 6 studies [20-25] met the inclusion criteria and were included in this systematic review.

The study identification and selection progression is summarized in Figure 1.

All studies were written in English. The 6 studies ranged in size from 44 to 234 patients for a total of 568 patients (Table 1). Three out of the studies were low quality studies according to Jadad's score (Table 1).

Clinical characteristics of the study populations are illustrated in the Table 1. All the trials included patients with an average age >50 years (Table 1). Compared to females, males were more frequently distributed in all trials (Table 1), except one, ranging from 48 to 82% (mean: 70.8%) in statin group and from 44 to 84% (mean: 65%) in control group.

Only two trials, conducted by Almroth et al. [21] and Negi et al. [24], were double-blind and placebo-controlled studies (Table 1).

Intervention treatment started from randomization day to 3 weeks before EC (Table 1).

Left atrial diameter ranged from 41 to 47 mm (mean: 44.6 mm) in statin group and from 41 to 46 mm (mean: 43.2 mm) in control group (Table 1).

The rate of previous coronary heart disease (CHD) was specified in four studies [20,21,24,25].

Statins used in the selected trials were: atorvastatin (at dosages ranging from 10 to 80 mg/day), pravastatin (40 mg/day) and rosuvastatin (20 mg/day) (Table 1).

The mean follow-up of the studies ranged from 30 days to 1 year (mean 3.7±4.1 months) (Table 1).

CHADS score [8] was reported only in one study [21].

Concomitant treatments and comorbidities are reported in Table 1.

Total cholesterol in the treated groups lowered from 15% to 32% according to the different type and dosage of statin (Table 1).

The rate of recurrences was different, according to the follow-up and to the treatment, among the studies ranging from as high as 66.6% (22/33 patients) to as low as 12.5% (3/24 patients) in statin groups and from as high as 83.8% (26/31 patients) to as low as 13.6% (3/22 patients) in control groups (Table 1).

**Figure 2:**
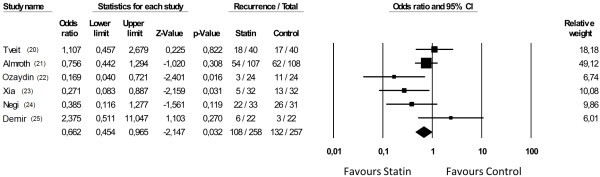
Effect of statins on recurrences of atrial fibrillation.

AF recurrence after EC occurred in 108/258 (41.8%) of patients treated with statins and in 132/257 (51.3%) controls. Statins significantly reduced the recurrences (O.R.: 0.662; 95% C.I., 0.45-0.96; p=0.03) (Figure 2) with an absolute risk reduction (ARR) of 0.095 and a number needed to treat (NNT) of 11. Heterogeneity across the studies [26] was moderate (I^2^=50, p=0.062).

## Discussion

This systematic review reports, for the first time, that upstream therapy with statins may be useful for the prevention of recurrence in patients with persistent AF who had undergone successful EC.

Previous meta-analyses studied the relationship between statins and AF, with inconclusive results. However they did not distinguish among the different types of AF (e.g. paroxysmal) [13-16], since observational studies [13] or cardiac surgical interventions, as coronary artery bypass grafting (CABG) [14,16], or patients undergone to EC [14,15]. Thus, these meta-analyses did not specifically address the question as to whether statin treatment is able to reduce or prevent recurrences in patients with persistent AF undergoing EC [13-16].

This issue may be clinically relevant as statins have anti-inflammatory properties and inhibit specific pathways generating oxidative stress, which are believed to trigger AF. Indeed, inflammation may interfere with the electrophysiological and structural changes of AF [27]. The presence of systemic inflammation, as shown by high serum levels of high-sensitivity C reactive protein (CRP) and interleukins 6 and 8, has been associated with AF [27]. Also, Dernellis et al. demonstrated that treatment with atorvastatin (initiated at a minimum of 20 mg/day and increased to a maximum of 40 mg/day) lowered CRP levels coincidentally with reduced recurrence in patients with paroxysmal AF [28]. Same results were detected by Negi et al. in atorvastatin (80 mg/day)- treated persistent AF patients, who underwent EC [24]. Oxidative stress plays also a pivotal role in inducing and maintaining AF [27,29]. The nicotinamide adenine dinucleotide phosphate (NADPH) oxidase is considered the most important cellular source of reactive oxygen species (ROS) in humans [30,31]; this enzyme is suggested to be implicated in functional changes of the atria which may favor AF [30]. Indeed, studies in humans have shown that myocytes from the right atrial appendage produce a significant amount of ROS through activation of NOX2, the catalytic subunit of NADPH oxidase [32]. Furthermore, NOX2 activation was observed in experimental models of AF [33] and in patients with post-surgery paroxysmal AF while it was within normal range in patients with permanent AF [33]. In accordance with this finding a serum activity of NOX2 was up-regulated only in patients with paroxysmal-persistent AF [34] and low serum levels of vitamin E, a known antioxidant [35], and associated with AF recurrence in patients who underwent EC [12]. As statin therapy inhibits ROS generation via down-regulation of NOX2 [33,36] and increase the serum levels of vitamin E [37], it would be tempting to speculate that inhibition of NOX2-generated ROS may hamper recurrence in patients with paroxysmal/persistent AF undergoing EC.

Therefore, down-regulation of NOX2 and inhibition of CRP may represent a plausible mechanism accounting for the beneficial effects observed by statin treatment in our meta-analysis. The analysis of six RCT demonstrated, in fact, an AF recurrence rate of 51.3% in control group and of 41.8% in statin-treated group with a relative risk reduction of 19%. The absolute risk reduction (9.5%) resulted in a number needed to treat of 11 patients with persistent AF.

This meta-analysis has the following limitations. Only two studies are placebo-controlled [21], [24] and all the remaining are open-label studies. The sample size of the included studies is low, ranging from 44 to 222 patients. The Jadad score, used to evaluate the quality of the trials, is low (<3) in about 50% of the studies included in the meta-analysis (Table 1). Furthermore, the female gender was less frequent with an average of 30% in the different studies. Finally, there is a great heterogeneity for cholesterol levels before and after treatment, statin type and dosage among the studies included in this analysis (Table 1).

## Conclusions

The results of this meta-analysis are of potential interest as they could represent a useful background to plan future studies to test the hypothesis that upstream therapy with statins may be beneficial to prevent AF recurrence in patients with persistent AF who undergo successful EC.

## Abbreviations

AF: Atrial fibrillation; EC: Electrical cardioversion; CHD: Coronary heart disease; RCTs: Randomized clinical trials; CRP: C reactive protein; NADPH: Nicotinamide adenine dinucleotide phosphate; ROS: Reactive oxygen species; CABG: Coronary artery bypass grafting.

## Competing interests

All the authors declare that they have no competing interests in relation to this manuscript.

## Authors’ contributions

All authors contributed to the conception and design of the study, participated in writing the manuscript, and approved the final draft. LL and LP undertook the literature search and retrieval of publications. LL and LP performed statistical analysis. All authors read and approved the final manuscript.

## Pre-publication history

The pre-publication history for this paper can be accessed here:

http://www.biomedcentral.com/1471-2261/12/107/prepub
